# Opinions of Medical Staff Regarding Antibiotic Resistance

**DOI:** 10.3390/antibiotics13060493

**Published:** 2024-05-27

**Authors:** Aneta Krolak-Ulińska, Piotr Merks, Urszula Religioni, Beata Chełstowska, Agnieszka Drab, Krystian Wdowiak, Katarzyna Plagens-Rotman, Zbigniew Doniec, Anna Staniszewska

**Affiliations:** 1 Anestesiology and Intensive Care Unit, Węgrów Regional Hospital, ul. Tadeusza Kościuszki 201, 07-100 Węgrów, Poland; anetau80@tlen.pl; 2 Faculty of Medicine, Collegium Medicum, Cardinal Stefan Wyszyński University, Wóycickiego 1/3, 01-938 Warsaw, Poland; 3 The Polish Pharmacy Practice Research Network (PPPRN), ul. Wóycickiego 1/3, 01-938 Warsaw, Poland; 4 School of Public Health, Centre of Postgraduate Medical Education of Warsaw, 01-824 Warsaw, Poland; 5 Department of Biochemistry and Laboratory Diagnostics, Faculty of Medicine, Collegium Medicum, Cardinal Stefan Wyszyński University, 01-938 Warsaw, Poland; 6 Department of Medical Informatics and Statistics with e-Health Laboratory, Medical University of Lublin, 20-059 Lublin, Poland; 7 Faculty of Medicine, Medical University of Lublin, 20-059 Lublin, Poland; 8 Center for Sexology and Pediatric, Adolescent Gynecology, Division of Gynecology, Department of Perinatology and Gynecology, Poznan University of Medical Sciences, 61-712 Poznan, Poland; 9 Pneumonology Clinic, Institute of Tuberculosis and Lung Diseases in Rabka-Zdrój, 34-700 Rabka-Zdrój, Poland; 10 Department of Pharmacology, Medical University of Warsaw, 01-079 Warsaw, Poland; anna.staniszewska@wum.edu.pl

**Keywords:** opinions, attitudes, knowledge, antibiotics, antibiotic resistance, medical staff

## Abstract

Introduction: Antibiotic resistance poses a significant threat to public health, that can lead to reduced effectiveness of many therapies, increased morbidity, longer hospitalization times, increased deaths, and additional costs for health care systems. Unreasonable use of antibiotics may result from a lack of adequate knowledge about antibiotic therapy and a lack of knowledge of the risks associated with antibiotic resistance, both among medical personnel and patients. Aim. The primary objective of the study was to verify the opinion of medical personnel on the risks associated with antibiotic resistance. Material and Methods: The study was conducted in 2023 among 605 Polish sanitary workers. An anonymous survey designed specifically for the purpose of the study was used. The survey was made available on the Internet through the Trade Unions of Pharmacy Workers and directly to hospitals with the support of local authorities. Results: The majority of respondents were women (77.36%). The largest group consisted of individuals over 40 years of age (55.04%). More than half of the respondents were nurses (56.20%), and every fourth of the respondents was a physician (23.64%). Most respondents consider antibiotic resistance to be a very serious (24.13%) or extremely serious (30.75%) problem. The problem of antibiotic resistance on a global scale was mentioned, especially in the opinions of physicians and nurses (*p* < 0.01), people working in the profession for over a year (*p* < 0.01), and people with a specialization or undergoing specialist training (*p* = 0.00). Similarly, these groups most often indicated that antibiotic resistance poses a problem in their workplace. The main problems of antibiotic resistance were the use of antibiotics in farm animals (36.69%), the pressure on patients to take antibiotics (38.84%), and the prophylactic use of antibiotics (43.15%). Conclusions: Medical personnel consider antibiotic resistance a somewhat serious problem, although not all agree in this regard. The risk of antibiotic resistance is much more seriously assessed by physicians and nurses, as well as by people with specializations or undergoing specialization training. Knowledge about antibiotic resistance should be further spread among all groups of medical personnel.

## 1. Introduction

Antibiotic resistance is one of the most significant threats to public health [1]. Antibiotics are a class of antimicrobials used to combat bacterial infections and antibiotic resistance, which is the most commonly used class of antimicrobials. Therefore, in order to resist forced environmental selection, bacteria tend to develop drug resistance, which leads to the ineffectiveness of previous therapies [2]. This situation in turn contributes to higher morbidity, longer hospitalizations, and higher mortality and generates many additional costs for healthcare systems [3,4,5]. It is estimated that in 2019, antibiotic resistance was the direct cause of 1.27 million deaths worldwide and contributed to another 4.95 million deaths [1]. In the European Union (EU) and the European Economic Area, 33,000 people die each year from infection with a resistant strain of bacteria, with no sign of change in the years to come. World Bank data indicate that AMR will contribute to the increase in healthcare costs by 1 trillion USD by 2050 and additional costs of lost productivity many times higher, translating into losses in GDP of up to 3.4 trillion USD per year [6]. The costs include, among others: patients’ stay in hospitals, which, in the case of hospitalization due to infections with a resistant strain of bacteria, lasts on average 13 days. Given the number of patients infected with drug-resistant bacteria, the annual hospitalization time amounts to 8 million days and costs up to 29,000 USD per patient [7]. Increased mass use of antibiotics during the COVID-19 pandemic will increase bacterial resistance and ultimately lead to more deaths [7]. 

The unreasonable use of antibiotics, which causes antibiotic resistance, is contributed by, among others, inadequate knowledge in the use of antibiotic therapy, unconsciousness of the risks associated with antibiotic resistance, lack of rapid and sufficient diagnostic tests, but also advertising of drugs and the pressure of patients to prescribe this group of drugs [8,9,10,11].

The optimal use of existing antimicrobials, the use of alternative treatment options, education of health care professionals and patients, the implementation of antibiotic policies, and effective measures to control infections are examples of strategies to prevent the development and spread of antibiotic resistance [2,12]. Due to their global scope, these issues have been recognized as priorities in the area of public health by a number of organizations and agencies around the world, including: the World Health Organization, the European Parliament, the European Center for Disease Prevention and Control (ECDC) for Disease Prevention and Control), the US Centers for Disease Control and Prevention (CDC), or the US Food and Drug Administration (FDA). In Poland, these types of activities are undertaken as part of the National Antibiotic Protection Program.

Awareness among medical personnel and patients of the dangers of antibiotic resistance is extremely important in the prevention of antibiotic resistance. Studies show that medical personnel are not always fully aware of the risks of the improper use of antibiotics [13,14]. The problem is also the lack of awareness of how medical personnel can contribute to reducing antibiotic resistance [15]. 

Due to the above, the main aim of our research was to explore the opinion of medical staff on the threats related to antibiotic resistance. In addition, the study sought a relationship between work experience and specialized training in antibiotic therapy and the awareness of respondents.

## 2. Material and Methods

### 2.1. Study Design

The study was conducted between September and December 2023 among 605 medical workers using an anonymous survey. It was an online survey where the link was provided to the pharmacists through Trade Unions of Pharmacy Workers (ZZPF—https://www.zzpf.org.pl (accessed on 1 April 2024)) and directly to hospitals through the Central Office of Marshal in Warsaw (https://mazovia.pl/en/ (accessed on 1 April 2024)) with the support of local governments. The questionnaire was sent with government support to all hospitals in the Masovian Voivodeship at the request of the management and sent to all medical employees.

The survey was created specifically for the purposes of the study and included 4 basic questions (containing specific questions) regarding the purpose of the study and the demographics (gender, age, profession, work experience, specialization, voivodeship, workplace, place of work: public/private hospital, nursery, individual practice, public/hospital pharmacy, and pharmaceutical company).

A questionnaire was created based on a literature review and our local needs due to the high impact of AMR in Poland.

The questionnaire consisted of four main questions as well as extension questions:

1. Rate on a scale of 1 (no problem) to 7 (very serious problem) how serious antibiotic resistance is in the following locations: worldwide/hospital in your city/in your province/your workplace.
2. Rate on a scale of 1 (no problem) to 7 (very serious problem) how strongly you think the following factors influence the increase in antibiotic resistance in Poland: antibiotic use inbred animals/antibiotic use in humans in your region/antibiotic use in patients in hospitals/patient pressure for a physician to prescribe antibiotics/prophylactic antibiotic use/antibiotic use in children.
3. Rate on a scale of 1 (no problem) to 7 (very serious problem) health care workers’ perception of the problem of drug resistance in the context of patient care and strategies to combat antibiotic resistance (questions only for respondents working in hospitals): the problem of antibiotic resistance affects patients under my care/rational use of antibiotics will reduce the problem of antibiotic resistance/antibiotics used incorrectly may worsen the patient’s health/prescribing antibiotics without indications is professionally unethical/limiting the prescription of antibiotics only in hospital treatment will help reduce the problem of antibiotic resistance/a policy of rational use of antibiotics should be introduced in my hospital/a computer application should be launched that would advise on the selection and duration of antibiotic therapy for patients in my hospital/a team should be established consisting of a physician specialist, clinical pharmacist and nurse providing personalized advice on antibiotic prescribing in my hospital/I will be happy to take part in any initiatives related to the use of antimicrobials in my hospital.
4. Rate on a scale of 1 (no problem) to 7 (very serious problem) the attitude of healthcare professionals towards strategies to combat antibiotic resistance: strategies to combat antibiotic resistance/regular hospital antibiotic audits and follow-up recommendations/limiting the prescribing of all antibiotics/limiting the prescribing of some antibiotics/easily accessible advice from microbiologists/regular educational training on the rational use of antibiotics.

Each of the questions respondents could rate on a scale of 1–7 (where 1—strongly disagree, 7—strongly agree).

### 2.2. Ethical-Legal Aspects

The study was approved by the Bioethics Commission of the Karol Marcinkowski Medical University in Poznań (Decision No. EC 988/23).

### 2.3. Statistical Analysis

The statistical analyses were performed using the STATISTICA data analysis software system version 13.0, StatSoft, Inc. (2017). https://www.statsoft.com (accessed on 1 April 2024).

The qualitative variables were presented with counts and percentages. To determine the dependence, strength, and direction between variables, the Pearson chi-square test and the Cramer V test were used. In all the calculations, a statistical significance of *p*  =  0.05 was used.

## 3. Results

### 3.1. Sample

In the study, 605 respondents participated, the majority of whom were women (77.36%). The largest group of respondents consisted of individuals over 40 years of age (55.04%). The vast majority of respondents live in the Masovian Voivodeship (79.50%). Nearly one-third of respondents live in a town with 50,000–100,000 inhabitants (34.21%), while one-fourth live in a town with 10,000–50,000 inhabitants (24.30%) (Table 1).

More than half of the respondents were nurses (56.20%), and every fourth of the respondents was a physician (23.64%). Nearly half of the respondents had been working in their profession for over 20 years (46.46%), and every fifth for 11–20 years (19.50%). The majority of respondents had a specialization (55.54%), while nearly one-sixth (14.71%) were working towards specialization. Most of the physicians and nurses worked in state hospitals (73.50%), while the pharmacists worked in public pharmacies (60.66%). Among nurses with a specialization (or prespecialization), the largest groups were surgical nurses (29.72%) and anesthetic and intensive care nurses (26.51%). Among the specialized physicians, specialists in internal medicine (12.77%) and general surgery (11.35%) were predominant, while among pharmacists with specialization, pharmacy pharmacists (64.44%) predominated (Table 2).

### 3.2. Opinions of Medical Practitioners

The majority of respondents considered antibiotic resistance to be a very serious (24.13%) or extremely serious (30.75%) problem on a global scale; however, on a closer scale (hospital in city/hospital in province/workplace), they primarily assess this problem as serious or somewhat serious (altogether, approximately 40% of the respondents) (Table 3).

The problem of antibiotic resistance on a global scale was perceived more seriously by:

- Physicians (more than other professions) and nurses (more than pharmacists) (*p* = 0.0003);
- Individuals working in the profession for more than a year (*p* < 0.01);
- Individuals with specialization or undergoing specialization training (*p* < 0.01). In all cases, there was a weak relationship.

The problem of antibiotic resistance on the scale of hospitals in a city was perceived more seriously by:

- Physicians (more than other professions) and nurses (more than pharmacists) (*p* < 0.01);
- Individuals working longer in the profession (*p* = 0.01);
- Individuals with specialization or undergoing specialization training (*p* < 0.01);
- Individuals working mainly in hospitals and clinics (*p* < 0.01) have weak relationships in all the above cases.

The problem of antibiotic resistance on the scale of hospitals in a province was perceived more seriously by:

- Physicians and nurses (*p* < 0.01);
- Individuals working longer in the profession (*p* < 0.01);
- Individuals with specialization or undergoing specialization training (*p* < 0.01);
- Individuals working in state hospitals (*p* < 0.01). In those cases, there were also weak relationships.

The problem of antibiotic resistance on the scale of the workplace (also with a rather weak relationship) was perceived more seriously by:

- Physicians and nurses (*p* < 0.01);
- Individuals working longer in the profession (*p* = 0.02);
- Individuals with specialization or undergoing specialization training (*p* < 0.01);
- Individuals working in state hospitals (*p* < 0.01).

The majority of respondents considered the usage of antibiotics in livestock (36.69%), patient pressure to receive antibiotics (38.84%), prophylactic use of antibiotics (43.15%), and usage of antibiotics in children (31.90%) as extremely serious problems in exacerbating antibiotic resistance in Poland. Meanwhile, the use of antibiotics by patients in provinces and hospitals was mainly classified as a serious problem or a very serious problem (altogether, this accounts for approximately 40% of the responses) (Table 4).

Usage of antibiotics in livestock in exacerbating antibiotic resistance in Poland was perceived more seriously by:

- Physicians (more than other professions) and nurses (more than pharmacists) (*p* < 0.01);
- Individuals with specialization or undergoing specialization training (*p* < 0.01), with weak relationships in all cases.

Usage of antibiotics by patients in the province in exacerbating antibiotic resistance in Poland was perceived more seriously (with a weak relationship) by:

- Physicians (more than other professions) and nurses (more than pharmacists) (*p* < 0.01);
- Individuals with specialization or undergoing specialization training (*p* < 0.01);
- Pharmacists working in hospitals and public pharmacies (*p* < 0.01).

Usage of antibiotics by patients in the hospital in exacerbating antibiotic resistance in Poland was perceived more seriously by:

- Physicians and nurses (*p* < 0.01);
- Individuals with specialization or undergoing specialization training (*p* < 0.01), with rather weak relationships in both cases.

Patient pressure to receive antibiotics in exacerbating antibiotic resistance in Poland was perceived more seriously (but with a weak relationship) by:

- Physicians and nurses (*p* < 0.01),
- Individuals with specialization or undergoing specialization training (*p* < 0.01).

Prophylactic use of antibiotics in exacerbating antibiotic resistance in Poland was perceived more seriously (with a weak relationship) by:

- Physicians and nurses (*p* < 0.01),
- Individuals with specialization or undergoing specialization training (*p* < 0.01),
- Pharmacists working in hospitals and public pharmacies (*p* = 0.03).

Usage of antibiotics in children in exacerbating antibiotic resistance in Poland was perceived more seriously by:

- Physicians and nurses (*p* < 0.01),
- Individuals with specialization or undergoing specialization training (*p* < 0.01),
- Pharmacists working in hospitals and public pharmacies (*p* = 0.01) and the relationships were rather weak in all cases.

Most respondents agreed with the statements that “improper use of antibiotics can worsen the patient’s health condition” and “prescribing antibiotics without indications is considered unprofessional conduct”. They also agree that “rational use of antibiotics will reduce the problem associated with antibiotic resistance”, and “limiting the prescription of antibiotics solely for hospital treatment will help reduce the problem of antibiotic resistance”. However, the agreement is more pronounced for the first two statements. Similar percentages of patients agree and disagree with the statement that “the issue of antibiotic resistance affects patients under my care” (41.2% vs. 43.74%).

With the statement “The issue of antibiotic resistance affects patients under my care”, those more likely to agree were:

- Physicians and nurses (*p* < 0.01),
- Individuals working longer in the profession (*p* = 0.01),
- Individuals with specialization or undergoing specialization training (*p* < 0.01), with a rather weak relationship in all cases.

Physicians (more than other professions) and nurses (more than pharmacists) (*p* < 0.01 in each case), as well as individuals with specialization or undergoing specialization training (*p* < 0.01 in each case), were more likely to agree with the remaining statements from Table 5—these are rather weak associations.

More respondents agreed (approximately 50%) than disagreed (approximately 40%) with the statements listed in Table 6; however, the popularity of positive over negative responses was small, ranging from a few to several percent.

With the statement “In my hospital, we should implement a policy for the rational use of antibiotics”, those more likely to agree were (weak relationships):

- Physicians and nurses (*p* < 0.01),
- Individuals with specialization or undergoing specialization training (*p* = 0.008).

With the statement “In my hospital, guidelines for the use of antibiotics should be implemented.”, those more likely to agree were:

- Physicians (more than other professions) and nurses (more than pharmacists) (*p* < 0.01), this is a weak relationship.

With the statement “In my hospital, there should be a team consisting of a specialist physician, a clinical pharmacist and a nurse providing personalized advice on prescribing antibiotics.”, those more likely to agree were:

- Physicians (more than other professions) and nurses (more than pharmacists) (*p* = 0.03), this is a weak relationship.
- Individuals with specialization or undergoing specialization training (*p* < 0.01), this is a rather weak relationship.

With the statement “I am willing to participate in any initiatives related to the use of antimicrobial agents in my hospital.”, those more likely to agree were:

- Physicians (*p* = 0.01), this is a weak relationship.

The majority of respondents agreed with the statements listed in Table 7 (around 50–60% of responses in favor vs. around 30% of responses against).

With the first four statements from Table 7, physicians (*p* < 0.01 with a weak relationship in each case) and individuals with specialization or undergoing specialization training (*p* < 0.01 in each case except the second question where *p* < 0.01; the first two associations are weak, the remaining two are rather weak) were more likely to agree.

With the remaining statements from Table 7, physicians (more than other professions) and nurses (more than pharmacists) (*p* = 0.03 and *p* < 0.01, these are rather weak associations) and individuals with specialization or undergoing specialization training (in both cases *p* < 0.01 these are weak associations) were more likely to agree.

## 4. Discussion

To the best of our knowledge, our study is the first of its kind to include such a large number of Polish medical workers. To the present day, research has been conducted on the awareness of antibiotic resistance among the general public [16,17] and medical students [18,19] in Poland. This makes it all the more important to study large groups of medical workers, which was the aim of this study. 

According to the results we obtained, the majority of respondents—Polish medical workers—consider antibiotic resistance a very serious problem (24.13%) or extremely serious (30.75%). The problem of antibiotic resistance on a global scale was mentioned, especially in the opinions of physicians and nurses, including people working in the profession for over a year, or people with specialization or undergoing specialist training. Similarly, these groups most often indicated that antibiotic resistance is a problem in their workplace.

Some publications directly indicate that a small number of studies, including those conducted in Europe, focus on AMR awareness among healthcare workers, although there are studies of this type conducted among social groups or students [20]. This type of research was conducted in Italy. As suggested by our study, Barchitta et al. (2021) indicated that Italian health workers have disparate knowledge and attitudes regarding antibiotic use and AMR awareness, stressing the need for educational and training interventions for specific professional groups [21]. Additionally, an Iranian study confirms large differences in the level of knowledge and approach to the use of antibiotics among health workers, indicating the need for the education of these groups [22]. However, Keizer et al. (2019), comparing health workers from Germany and the Netherlands, indicate a fairly large and similar awareness of different groups, although German workers, compared to Dutch, see more possibilities of influencing rationalizations of antibiotic therapy [15]. 

Studies of AMR awareness of individual professional groups, e.g., nurses, also do not give unambiguous results. Nurses demonstrate moderate awareness of the AMR problem and, importantly, this awareness is not dependent on demographic characteristics or their attitudes and general knowledge. There was also no link between awareness and the total number of years of experience or specialist training [23,24]. In turn, a study of young Italian physicians showed that their knowledge of antibiotic therapy was low compared to the declared one [25].

There are also few studies involving pharmacists, although it is stressed that the broader role of this professional group, including the provision of various patient care services, results in better patient health outcomes and lower healthcare costs. Properly trained pharmacists can therefore have a significant impact on increasing rational antibiotic use, which in turn can affect the global problem of antimicrobial resistance [26,27]. Moreover, pharmacists can be the right group of professionals to raise awareness. A study assessing the pharmaceutical intervention in increasing patient knowledge of antibiotic therapy shows that those who took the advice of a local pharmacist showed much better knowledge of antibiotic use [28].

The results obtained in our study indicate that approximately half of respondents indicate the importance of the AMR problem. This data is of particular importance due to the fact that the AMR problem is indicated as one of the most important public health problems by many organizations, including WHO [1]. WHO highlights the scale of improper use of antimicrobials as a significant risk factor, often in improper doses and sometimes in the case of nonbacterial infections. The evolution of bacterial resistance as a result of the widespread and irrational use of antibiotics poses serious challenges to healthcare systems and increases the cost of treatment [29,30,31]. Given the high frequency of prescribing antibiotics and the growing global consumption of antibiotics, there is an urgent need to address this problem in order to protect public health [32,33].

To address the challenges of improper antibiotic therapy, various interventions are recommended, including the promotion, monitoring and evaluation of the rational use of antibiotic therapy at various levels of healthcare, as well as the adoption of clinical guidelines or the establishment of drug and therapeutic committees. For this purpose, it is important to include training in rational pharmacotherapy in educational programs, as well as the continuous education of medical staff and the public on antibiotic therapy [7,34,35].

The role of health workers is crucial in shaping public awareness of rational antibiotic therapy [7]. The primary objective of rational management of antimicrobials should be to improve patient outcomes while minimizing the medical and economic impacts of antibiotics. Given the significant differences in awareness of AMR among different groups of health workers, it is essential to plan educational interventions aimed at specific target groups.

## 5. Conclusions

The unreasonable use of antibiotics is a worldwide public health threat. Many factors contribute to an increase in antibiotic resistance, but one of the biggest is insufficient awareness about the risks associated with AMR both among medical professionals and the public. This situation has both medical consequences, leading to significant morbidity and mortality, and financial consequences for health systems and economies.

In this context, it is crucial to take targeted action as soon as possible, aiming not only at monitoring and monitoring the situation, but also to educate groups on the risks associated with antibiotic resistance. Only intentional, long-term action can bring expected results, including the rationalization of the use of this group of drugs.

## Figures and Tables

**Table 1:** Demographic characteristics of the study group.

	Study Group (*n* = 605)
**Sex**	
Women	468 (77.36%)
Men	124 (20.50%)
No response	13 (2.14%)
**Age**	
<25 years	28 (4.63%)
25–30 years	77 (12.73%)
31–35 years	82 (13.55%)
36–40 years	85 (14.05%)
>40 years	333 (55.04%)
**Size of place of residence**	
Village	24 (3.97%)
Town up to 10,000 inhabitants	26 (4.30%)
Town 10,000–50,000 inhabitants	147 (24.30%)
Town 50,000–100,000 inhabitants	207 (34.21%)
Town 100,000–500,000 inhabitants	113 (18.68%)
City above 500,000 inhabitants	88 (14.54%)

**Table 2:** Professional characteristics of respondents.

	Study Group (*n* = 605)
**Job**	
Nurse	340 (56.20%)
Physician	143 (23.64%)
Pharmacist	122 (20.16%)
**Length of employment**	
<1 years	14 (2.31%)
1–5 years	103 (17.02%)
6–10 years	89 (14.71%)
11–20 years	118 (19.50%)
>20 years	281 (46.46%)
**Specialization**	
Yes	336 (55.54%)
No	180 (29.75%)
In progress	89 (14.71%)
**Main workplace of physicians and nurses (*n* = 483)**	
State hospital	355 (73.50%)
Outpatient clinic	53 (10.97%%)
Private hospital	48 (9.94%)
Private practice	17 (3.52%%)
Other	10 (2.07%)
**Main workplace of pharmacists (*n* = 122)**	
Hospital pharmacy	25 (20.49%)
Public pharmacy	74 (60.66%)
Pharmaceutical company	13 (10.66%)
Other	10 (8.19%)

**Table 3:** Responses of the respondents regarding the seriousness of the issue of antibiotic resistance on different scales.

	How Significant an Issue Is Antibiotic Resistance on the Scale…			
	Worldwide	Hospitals in Your City	Hospitals in Your Province	Your Workplace
No problem	2 (0.33%)	25 (4.13%)	26 (4.30%)	37 (6.12%)
Minor problem	29 (4.79%)	58 (9.59%)	64 (10.58%)	91 (15.04%)
Moderate problem	66 (10.91%)	94 (15.54%)	78 (12.89%)	94 (15.54%)
Somewhat serious problem	90 (14.88%)	139 (22.98%)	135 (22.31%)	113 (18.68%)
Serious problem	86 (14.21%)	115 (19.00%)	113 (18.68%)	109 (18.02%)
Very serious problem	146 (24.13%)	81 (13.39%)	88 (14.55%)	72 (11.90%)
Extremely serious problem	186 (30.75%)	93 (15.37%)	101 (16.69%)	89 (14.70%)

**Table 4:** Responses to questions regarding the exacerbation of antibiotic resistance through selected antibiotic usage methods.

	How Significant of an Issue Is… in Exacerbating Antibiotic Resistance in Poland?					
	Usage of Antibiotics in Livestock	Usage of Antibiotics by Patients in Province	Usage of Antibiotics by Patients in the Hospital	Patient Pressure to Receive Antibiotics	Prophylactic Use of Antibiotics	Usage of Antibiotics in Children
No problem	15 (2.48%)	11 (1.82%)	15 (2.48%)	17 (2.81%)	12 (1.98%)	16 (2.64%)
Minor problem	42 (6.94%)	58 (9.59%)	57 (9.42%)	44 (7.27%)	56 (9.25%)	50 (8.26%)
Moderate problem	73 (12.07%)	60 (9.92%)	84 (13.88%)	51 (8.43%)	54 (8.93%)	56 (9.26%)
Somewhat serious problem	68 (11.24%)	98 (16.20%)	85 (14.05%)	55 (9.09%)	52 (8.59%)	73 (12.07%)
Serious problem	83 (13.72%)	123 (20.33%)	146 (24.13%)	79 (13.06%)	77 (12.73%)	100 (16.53%)
Very serious problem	102 (16.86%)	136 (22.48%)	112 (18.51%)	124 (20.50%)	93 (15.37%)	117 (19.34%)
Extremely serious problem	222 (36.69%)	119 (19.66%)	106 (17.53%)	235 (38.84%)	261 (43.15%)	193 (31.90%)

**Table 5:** Perception of antibiotic resistance among hospital staff (*n* = 432) in the context of patient care and strategies to combat antibiotic resistance. Part 1.

	To What Extent Do You Agree with the Statement				
	The Issue of Antibiotic Resistance Affects Patients Under My Care.	Rational Use of Antibiotics Will Reduce the Problem Associated with Antibiotic Resistance.	Improper Use of Antibiotics Can Worsen the Patient’s Health Condition.	Prescribing Antibiotics without Indications Is Considered Unprofessional Conduct.	Limiting the Prescription of Antibiotics Solely for Hospital Treatment Will Help Reduce the Problem of Antibiotic Resistance.
Strongly disagree	44 (7.99%)	25 (4.54%)	12 (2.18%)	13 (2.36%)	28 (5.13%)
Disagree	95 (17.24%)	73 (13.25%)	50 (9.07%)	48 (8.72%)	74 (13.55%)
Slightly disagree	102 (18.51%)	73 (13.25%)	52 (9.44%)	58 (10.55%)	72 (13.19%)
Neutral	83 (15.06%)	68 (12.34%)	43 (7.80%)	25 (4.55%)	73 (13.37%)
Slightly agree	84 (15.25%)	92 (16.70%)	74 (13.43%)	60 (10.91%)	90 (16.48%)
Agree	69 (12.52%)	88 (15.97%)	123 (22.32%)	101 (18.36%)	82 (15.02%)
Strongly agree	74 (13.43%)	132 (23.95%)	197 (35.76%)	245 (44.55%)	127 (23.26%)

**Table 6:** Perception of antibiotic resistance among hospital staff (*n* = 432) in the context of patient care and strategies to combat antibiotic resistance. Part 2.

	To What Extent Do You Agree with the Statement				
	In My Hospital, We Should Implement a Policy for the Rational Use of Antibiotics.	In My Hospital, Guidelines for the Use of Antibiotics Should Be Implemented.	In My Hospital, a Computer Application Should Be Launched to Provide Advice on the Selection and Duration of Antibiotic Therapy for Patients.	In My Hospital, There Should Be a Team Consisting of a Specialist Physician, Clinical Pharmacist, and Nurse Providing Personalized Advice on Antibiotic Prescribing.	I Am Willing to Participate in Any Initiatives Related to the Use of Antimicrobial Agents in My Hospital.
Strongly disagree	37 (8.56%)	39 (9.18%)	34 (7.89%)	56 (13.11%)	47 (11.01%)
Disagree	70 (16.20%)	64 (15.06%)	62 (14.39%)	56 (13.11%)	54 (12.65%)
Slightly disagree	65 (15.05%)	56 (13.18%)	53 (12.29%)	47 (11.01%)	58 (13.58%)
Neutral	47 (10.88%)	51 (12.00%)	46 (10.67%)	60 (14.05%)	62 (14.52%)
Slightly agree	61 (14.12%)	65 (15.29%)	63 (14.62%)	44 (10.30%)	54 (12.65%)
Agree	56 (12.96%)	56 (13.18%)	68 (15.78%)	68 (15.93%)	60 (14.05%)
Strongly agree	96 (22.23%)	94 (22.11%)	105 (24.36%)	96 (22.49%)	92 (21.54%)

**Table 7:** Healthcare workers’ approach to strategies aimed at combating antibiotic resistance.

	To What Extent Do You Agree with the Statement					
	Strategies to Combat Antibiotic Resistance Can Help Limit This Phenomenon.	Regular Audits of Antibiotic Therapy in Hospitals along with Postaudit Recommendations Can Help Reduce Antibiotic Resistance.	Limiting the Prescription of All Antibiotics Can Reduce Antibiotic Resistance.	Limiting the Prescription of Certain Antibiotics Can Reduce Antibiotic Resistance.	Easily Accessible Advice from Microbiologists Can Help Reduce Antibiotic Resistance.	Regular Educational Training on the Rational Use of Antibiotics Can Reduce Antibiotic Resistance.
Strongly disagree	23 (3.80%)	39 (6.45%)	28 (4.63%)	27 (4.46%)	33 (5.45%)	31 (5.12%)
Disagree	58 (9.59%)	71 (11.74%)	76 (12.56%)	68 (11.24%)	65 (10.74%)	67 (11.07%)
Slightly disagree	97 (16.03%)	91 (15.04%)	76 (12.56%)	84 (13.88%)	73 (12.07%)	84 (13.88%)
Neutral	89 (14.71%)	75 (12.39%)	90 (14.88%)	74 (12.23%)	59 (9.75%)	62 (10.25%)
Slightly agree	80 (13.22%)	86 (14.21%)	91 (15.04%)	102 (16.86%)	80 (13.22%)	74 (12.23%)
Agree	138 (22.81%)	136 (22.48%)	118 (19.50%)	114 (18.84%)	126 (20.83%)	123 (20.33%)
Strongly agree	120 (19.84%)	107 (17.69%)	126 (20.83%)	136 (22.49%)	169 (27.94%)	164 (27.12%)

## Data Availability

All data are available from the corresponding author.

## Abbreviations

AMR	antimicrobial resistance
AMS	antimicrobial stewardship
ASP	antibiotic stewardship programs
EU	European Union
WHO	World Health Organization
